# *Massilia varians* P2-4: a potential biocontrol agent against pathogenic *Pseudomonas aeruginosa* in *Eriocheir sinensis*

**DOI:** 10.3389/fmicb.2026.1863139

**Published:** 2026-06-30

**Authors:** Yiyao Liu, Yueqi Yang, Meiling Liu, Sijia Chen, Haipeng Cao, Chunlei Gai, Weidong Ye

**Affiliations:** 1 National Pathogen Collection Center for Aquatic Animals, National Demonstration Center for Experimental Fisheries Science Education, Shanghai Ocean University, Shanghai, China; 2 Key Laboratory of Freshwater Fishery Germplasm Resources, Ministry of Agriculture and Rural Affairs of China, Shanghai, China; 3 Department of Clinical Laboratory, Quzhou Affiliated Hospital of Wenzhou Medical University, Quzhou, China; 4 Marine Science Research Institute of Shandong Province (National Oceanographic Center, Qingdao), Qingdao, China

**Keywords:** antibacterial potential, characterization, crab, *Massilia varians*, *Pseudomonas aeruginosa*

## Abstract

*Pseudomonas aeruginosa* is a clinically significant bacterial pathogen that poses a serious threat to aquaculture. However, there is limited information on *Massilia* isolates against pathogenic *P. aeruginosa* in aquaculture. In this study, a potential predator, *M. varians* isolate P2-4, was isolated from aquaculture sediment using Chinese mitten crab *Eriocheir sinensis*-pathogenic *P. aeruginosa* as the prey bacterium, and its genomic features, antibacterial-related genes, virulence genes, toxic metabolites, pathogenicity, antibacterial spectrum, and *in vitro* and *in vivo* antibacterial effects against pathogenic *P. aeruginosa* in *E. sinensis* were further characterized. Isolate P2-4 possessed one chromosome and one plasmid, with a total of 75 tRNAs, 7 5S rRNAs, 7 16S rRNAs, 7 23S rRNAs, 34 sRNAs, 5,238 coding genes, 20 genomic islands, 1 prophage, 23 insertion sequences, and 102 repeat sequences. It harbored 19 antibacterial-related genes (*pilA*, *pilB*, *pilC*, *pilD*, *pilF*, *pilG*, *pilH*, *pilM*, *pilO*, *pilP*, *pilQ*, *pilS*, *pilR*, *pilT*, *mltA*, *mltB*, *mltC*, *mltD*, and *dacB*) associated with cellular motility and cell wall lysis. In addition, the isolate carried no virulence genes, was unable to produce hemolysin, hydrogen sulfide, nitrite, and ammonia, and was avirulent in *E. sinensis* with a 7-day acute LD_50_ value of above 5.0 × 10^8^ CFU/mL. Furthermore, the isolate exerted potential antibacterial activity against pathogenic *Shewanella algae*, *Aeromonas caviae*, *A. hydrophila*, and *Photobacterium damselae*, besides *P. aeruginosa*. It exhibited bacteriostatic rates of 99.35 to 99.99% against pathogenic *P. aeruginosa* at concentrations of 1.0 × 10^3^ to 1.0 × 10^5^ CFU/mL, and displayed relative percentage survivals of 42.31 to 73.08% against *P. aeruginosa* infection in *E. sinensis* at doses of 6.0 × 10^3^ to 6.0 × 10^5^ CFU/g diet. To our knowledge, this study, for the first time, demonstrates a *M. varians* strain as a potential biocontrol agent against pathogenic *P. aeruginosa* in aquaculture.

## 1 Introduction

*Pseudomonas aeruginosa* is a Gram-negative zoonotic bacterium that is widely distributed in diverse ecological environments, such as soil, water, and sewage (Liao et al., 2022). This bacterium can result in gill necrosis in *Oziotelphusa senex*, hemorrhagic septicemia in *Oreochromis niloticus*, and shell erosion disease in *Bellamya aeruginosa* (Devakumar et al., 2013; El-Tarabili et al., 2023; Zhang et al., 2025). Currently, control of *P. aeruginosa* infections in aquaculture relies heavily on antibiotics (Ali et al., 2021; Kholil et al., 2015). However, frequent use of antibiotics increases the risk of antimicrobial resistance and may disrupt the aquatic ecological balance (Frascaroli et al., 2024; Pepi and Focardi, 2021). Thus, environmentally friendly control agents should be urgently explored to mitigate *P. aeruginosa* infections in aquaculture.

Predatory bacteria are a diverse group of Gram-negative microorganisms that have the capacity to prey on or inhibit other bacteria by secreting lytic proteins and lysing cell walls (Novick, 2021; Kamada et al., 2023; Mohsenipour et al., 2024). They are grouped into non-obligate predators (prey-independent growth on organic nutrients) and obligate predators (predatory growth on living prey cells) (Bauer and Forchhammer, 2021). Currently, obligate predators, such as *Bdellovibrio* and *Halobacteriovorax,* exhibit remarkable inhibition against pathogenic *Acinetobacter venetianus*, *Aeromonas hydrophila*, *A. veronii*, *Proteus penneri*, *Vibrio cholerae*, *V. parahaemolyticus*, and *V. vulnificus* in aquaculture (Cao et al., 2012; Cao et al., 2014a; Cao et al., 2014b; Cao et al., 2015; Cao et al., 2019; Huang et al., 2020; Ottaviani et al., 2018) and provide significant protection of aquaculture animals against *A. veronii* and *Shewanella putrefaciens* infections (Liu et al., 2022; Liu et al., 2024). However, scarce information is available on non-obligate predators against *P. aeruginosa* in aquaculture.

*Massilia* is a genus of Gram-negative bacteria distributed in a wide range of environments, such as soil, aquatic systems, and the surfaces of various organisms (Xu et al., 2023). Although the role of *Massilia* remains controversial due to occasional cases of human septicemia and osteomyelitis caused by a few isolates (Lindquist et al., 2003), it is still recognized as a promising probiotic with remarkable antibacterial activity (Yang et al., 2019). Several *Massilia* strains display potent antibacterial capability against multiple pathogens, such as *Ralstonia solanacearum*, *Curtobacterium flacumfaciens*, and *Paenarthrobacter ilicis* (Li et al., 2021; Sedláček et al., 2025). Nevertheless, there is currently no documented evidence suggesting *Massilia* strains as potential predators against *P. aeruginosa* in aquaculture.

Chinese mitten crab *E. sinensis* is an economically important aquatic product in East and Southeast Asian countries (Agbekpornu et al., 2018). However, the aquaculture of this crab has recently been severely affected by *P. aeruginosa* (He et al., 2026). In this study, *M. varians* isolate P2-4, a potential predator against *E. sinensis*-pathogenic *P. aeruginosa*, was isolated from aquaculture sediment, and its genomic features, antibacterial-related genes, virulence genes, toxic metabolites, pathogenicity, antibacterial spectrum, and *in vitro* and *in vivo* antibacterial effects against pathogenic *P. aeruginosa* were further characterized. To our knowledge, this is the first study to identify a *M. varians* strain as a potential biocontrol agent against pathogenic *P. aeruginosa* in aquaculture.

## 2 Materials and methods

### 2.1 Animal ethics

All animal experiments and experimental protocols were approved by the Institutional Animal Care and Use Ethics Committee of Shanghai Ocean University under approval no. SHOU-DW-2025-050.

### 2.2 Isolation of potential predatory isolate

The isolation of a potential predatory isolate was carried out according to Wang (2020). Prior to isolation, pathogenic *P. aeruginosa* strain HX-1, isolated from diseased *E. sinensis* suffering from hepatopancreas necrosis disease and identified via genome sequencing and phenotypic methods (He et al., 2026), was obtained from the National Pathogen Collection Center for Aquatic Animals in Shanghai, China. A fresh suspension of *P. aeruginosa* strain HX-1 was prepared by culturing the strain in nutrient broth (NB; Sinopharm Chemical Reagent Co., Ltd., Shanghai, China) for 12 h, followed by centrifugation at 4000 r/min for 10 min and resuspension in normal saline (Wingender et al., 2001). After serial ten-fold dilution with sterile normal saline, the suspension was quantified to 5.0 × 10^9^ colony-forming units (CFU)/ml on nutrient agar (NA, Qingdao Hope Bio-Technology Co., Ltd., Shandong, China) plates (Rodgers et al., 2023). The aquaculture sediment (1.0 g), collected from an aquaculture farm in Shandong, China, was suspended in 9.0 mL of sterile normal saline and vortexed at 30 °C for 30 min to achieve a mixture. The mixture was then subjected to serial ten-fold dilution with sterile normal saline. Subsequently, these dilutions were examined for the presence of plaques using the double-layer agar plate method (Ottaviani et al., 2020), employing *P. aeruginosa* strain HX-1 as prey. Briefly, 100 μL of the dilution and 200 μL of freshly prepared *P. aeruginosa* suspension were added to 10 mL of 0.6% agar melted and kept at 55 °C, mixed, and poured onto a pre-prepared 1.5% agar plate until solid. The plaques were observed carefully after incubation of the double-layer plates at 30 °C for 5 days. Aliquots (0.2 mL) from plaque-positive dilutions were then spread onto NA plates and incubated at 30 °C for 24 h. Individual colonies were picked using a flame-sterilized inoculating loop and purified through repeated streaking and subculturing on NA plates under identical incubation conditions. The purified isolates were further assessed for plaque-forming ability using the double-layer agar plate method (Ottaviani et al., 2020), with *P. aeruginosa* strain HX-1 as prey to confirm predatory activity. Finally, the potential predatory isolate was cultured on NA slants at 30 °C for 24 h prior to storage at 4 °C.

### 2.3 Identification of potential predatory isolate

#### 2.3.1 16S rRNA gene sequencing analysis

The genomic DNA of the isolate was extracted using a TIANamp bacteria DNA kit (Tiangen Biotech Co., Ltd., Beijing, China). The 16S rRNA gene of the isolate was amplified via PCR with universal primers 27F (5’-AGAGTTTGATCCTGGCTCAG-3′) and 1492R (5’-GGTTACCTTGTTACGACTT-3’), as recommended by Tian et al. (2023). The PCR reaction mixture consisted of 10 μM of each primer (1 μL), 2 × Taq Master mix (Vazyme, Nanjing, China) (20 μL), 20 ng/μL of template DNA (1 μL), and ddH_2_O (17 μL). The PCR amplification conditions included initial denaturation at 95 °C for 3 min, followed by 35 cycles of denaturation at 95 °C for 1 min, annealing at 60 °C for 1 min, and extension at 72 °C for 1 min, with a final extension at 72 °C for 10 min. The resultant PCR product was subjected to 1.0% (w/v) agarose gel electrophoresis and was sequenced by Shanghai MAP Biotech. Co., Ltd. in Shanghai, China. The obtained sequence was subjected to homology analysis using the Basic Local Alignment Search Tool (BLAST)^1^ against the GenBank database. Phylogenetic analysis was conducted using the neighbor-joining method in MEGA 11.0 software (Pennsylvania State University, Pennsylvania, United States).

#### 2.3.2 Genomic sequencing analysis

Whole genome sequencing of the isolate was conducted at a sequencing depth of 96x using the Illumina HiSeq and PacBio platforms (Shanghai Majorbio Biopharm Technology Co., Ltd., Shanghai, China), according to Li et al. (2023). Sequencing reads were assembled using SOAPdenovo2 and CANU software (Luo et al., 2012; Koren et al., 2017). Concurrently, the construction of circular genome maps was undertaken with the use of Circos (v. 0.69) (Krzywinski et al., 2009). A comprehensive analysis of various genomic features, such as Genome size (bp), GC average content, gene average length, repeated regions, number of chromosomes and plasmids, tRNA, rRNA, sRNA, coding sequences (CDS), tandem repeats, prophages, genomic islands (GI), and insertion sequences (IS), were predicted using NextDenovo (v. 2.5.2), RepeatMasker (v. 4.1.4), tRNAscan-SE (v. 2.0.12), Barrnap (v. 0.9), Infernal (v. 1.1.5), Glimmer (v. 3.02), GeneMarkS (v. 4.3), Tandem Repeats Finder (v. 4.07), Phagaro (v. 2.3.0), IslandPath-DIMOB (v. 1.0.0), and ISEScan (v. 1.7.2.1) software (Benson, 1999; Besemer and Borodovsky, 2005; Chan and Lowe, 2019; Fouts, 2006; Xie and Tang, 2017). Overall genome-relatedness indices between the isolate and closely related bacterial species, such as average nucleotide identity (ANI) and digital DNA–DNA hybridization (dDDH), were calculated using MUMmer (v. 3.23) and Genome-to-Genome Distance Calculator (v. 3.0) software (Ribeiro et al., 2021). Species boundaries were defined based on the thresholds of 95% ANI and 70% dDDH (Candeliere et al., 2021).

#### 2.3.3 Phenotypic identification

After Gram staining observation and cultivation on thiosulfate–citrate–bile salts–sucrose (TCBS; Qingdao Hope Bio-Technology Co., Ltd., Shandong, China) agar, the phenotypic characterization of the isolate was conducted in triplicate using API 20NE test strips (bioMérieux, Marcy-l’Étoile, France), strictly in accordance with the manufacturer’s instructions (Roxo et al., 2025). The phenotypic traits of the isolate were compared with those previously described (Cho et al., 2017; Chaudhary and Kim, 2017; Weon et al., 2010).

### 2.4 Virulence and antibacterial-related gene assay

The isolate genome (obtained in section 2.3.2) was subjected to BLAST analysis against the virulence factors database^2^ to identify virulence genes (Liu et al., 2019). Meanwhile, Clusters of Orthologous Genes (COG, v. 2020.06) and Kyoto Encyclopedia of Genes and Genomes (KEGG, v. 20,230,830) analyses in the isolate genome were conducted to identify antibacterial-related genes described by Kanehisa and Goto (2000) using BLAST2GO (v. 1.0) and DIAMOND blastp (v. 2.0.14) software (Tatusov et al., 2000; Kanehisa, 2000). The cutoff values were set at E-value ≤ 1e^−5^, identity ≥ 90%, and coverage ≥ 90% (Wang et al., 2021).

### 2.5 Toxic metabolite production assay

#### 2.5.1 Hemolysin production assay

The hemolysin production of the isolate was examined in triplicate with rabbit blood agar (RBA) plates according to Gai et al. (2023). Briefly, the isolate was inoculated onto RBA plates (Qingdao Hope Bio-Technology Co., Ltd., Shandong, China) and incubated at 30 °C for 48 h to observe for hemolytic zones surrounding bacterial colonies. An incomplete transparent zone indicated *α*-hemolysin, whereas a clear colorless zone indicated *β*-hemolysin (Castro-Escarpulli et al., 2003). *Rhodobacter azotoformans* strain SY5, an avirulent strain for *E. sinensis* isolated from aquaculture sediment and identified via molecular and phenotypic methods (Cao et al., 2022; Cao et al., 2023), was used as the control.

#### 2.5.2 Hydrogen sulfide, nitrite, and ammonia production assay

The isolate was examined in triplicate for hydrogen sulfide, nitrite, and ammonia production using microbiochemical identification tubes, as recommended by Kang et al. (2019). Briefly, the isolate was inoculated into the hydrogen sulfide, nitrite, and ammonia biochemical identification tubes following the manufacturer’s guidelines (Qingdao Hope Bio-Technology Co., Ltd., Shandong, China) and incubated at 37 °C for 24 h. The production of hydrogen sulfide, nitrite, and ammonia was determined based on color changes within tubes following the manufacturer’s instructions. *R. azotoformans* strain SY5 served as the control.

### 2.6 Antibacterial spectrum assay

Prior to this assay, the isolate was inoculated into NB for 24 h of culture at 30 °C. The culture was harvested by centrifugation at 4000 r/min for 10 min, and the resulting cell pellet was resuspended in normal saline to prepare the isolate suspension. Thirteen pathogenic strains were obtained from the National Pathogen Collection Center for Aquatic Animals in Shanghai, China, such as *P. aeruginosa* strains FJ1-2, FJ1-5, FJ1-9, FJ3-2, and PX3-3; *S. algae* strains RZ2-2, RZ3-4, and RZ2-1; *A. caviae* strains PX2-5 and PX3-1; *A. hydrophila* strains PX2-8 and PX2-6; and *Photobacterium damselae* strain YJ-1. Suspensions of these pathogenic strains were freshly prepared following the method described in section 2.2. The cell densities of the isolate and pathogenic strain suspensions were quantified by counting CFU on NA plates through serial 10-fold dilution in sterile normal saline. The predatory activity of the isolate against the 13 pathogenic strains was examined in triplicate using the double-layer agar plate method (Ottaviani et al., 2020). Briefly, 50 μL of the isolate suspension (1.0 × 10^6^ CFU/mL) and 100 μL of each pathogenic strain suspension (5.0 × 10^9^ CFU/mL) were added to 10 mL of 0.6% agar melted and kept at 55 °C, mixed, and poured onto a pre-prepared 1.5% agar plate until solid. Following incubation at 30 °C for 48 h, the double-layer agar plates were observed for the presence of plaques, and plaque-forming unit (PFU) counts were recorded. The predatory activity of the isolate against *P. aeruginosa* strain HX-1 under identical conditions was tested as the control.

### 2.7 *In vitro* antibacterial effect against *Pseudomonas aeruginosa* assay

The *in vitro* antibacterial effect of the isolate against *P. aeruginosa* was examined in 12,250-mL glass flasks, allocated to one control and three treatment groups, with three flasks per group. Prior to this assay, suspensions of the isolate and *P. aeruginosa* strain HX-1 were freshly prepared following the method described in sections 2.2 and 2.6. Following quantification through CFU counting on NA plates after serial 10-fold dilutions with sterile normal saline, suspensions of the isolate and *P. aeruginosa* were, respectively, introduced into the treatment flasks, followed by the addition of filtered farming water (prepared and autoclaved as described by Cao et al. (2019)) to reach a final volume of 200 mL, resulting in cell densities of 1.0 × 10^3^, 1.0 × 10^4^, and 1.0 × 10^5^ CFU/mL for the isolate, and a constant cell density of 1.0 × 10^5^ for *P. aeruginosa.* The mixtures were subsequently incubated at 30 °C with shaking at 180 r/min for 5 days. The control flasks containing only *P. aeruginosa* received the same treatment as described above. Cell densities of *P. aeruginosa* in the control and treatment groups were measured daily according to Cao et al. (2015), using CFU counting on TCBS agar plates as recommended by Hidayati (2009) through serial 10-fold dilution in sterile normal saline (Kim et al., 2012). The bacteriostatic rate (%) was calculated according to the following formula (Zhang et al., 2022a).

Where N_C_ represents the cell density in the control group, and N indicates the cell densities in the treatment groups.

### 2.8 Bacterial pathogenicity and protective effect assay

#### 2.8.1 Experimental crabs

Healthy *E. sinensis* (22.12 ± 0.6 g in weight) were provided by Nantong Duoruixian E-commerce Co., Ltd. in Jiangsu, China. Following careful health assessment through physical observation and pathogen examination of 10 individuals according to Ding et al. (2015) and Yang et al. (2018), the experimental crabs were acclimated in aerated tap water with pH7-8, dissolved-oxygen ≥ 6.0 mg/L, and total ammonia ≤ 0.2 mg/L at 28 °C under a 12-h light:12-h dark cycle for 14 days (Speights and McCoy, 2017; Xu et al., 2025).

#### 2.8.2 Bacterial pathogenicity assay

The pathogenicity of the isolate was examined by testing the median lethal dose (LD_50_) toward *E. sinensis* according to Cao et al. (2023). Prior to the LD_50_ test, the isolate suspension was freshly prepared as described above (see section 2.6), and serially 10-fold diluted in normal saline to achieve cell densities of 5.0 × 10^4^, 5.0 × 10^5^, 5.0 × 10^6^, 5.0 × 10^7^, and 5.0 × 10^8^ CFU/mL, which were quantified through CFU counting on NA agar plates after serial 10-fold dilutions with sterile normal saline. The LD_50_ test was carried out in 18 glass aquaria (60 cm × 40 cm × 40 cm). Each aquarium served as an experimental unit and contained 10 crabs with six tiles at the bottom and 10 L of aerated tap water at 28 °C. The aquaria were randomly divided into one control and five treatment groups, with three replicate aquaria per group. Crabs in the five treatment groups were, respectively, injected with 0.1 mL of the isolate suspension (5.0 × 10^4^, 5.0 × 10^5^, 5.0 × 10^6^, 5.0 × 10^7^, and 5.0 × 10^8^ CFU/mL) at the base of the third periopod. In contrast, the control crabs received an injection, with 0.1 mL of sterile normal saline. During the 7-day test period, crabs were kept at 28 °C under a 12-h light:12-h dark cycle (Speights and McCoy, 2017), and were fed sterile-processed basal diet (with the formulation and proximate composition described previously (Cao et al., 2022); Hongxiang Feed Biotech. Co., Ltd., Jiangsu, China) at 3% of the total body weight up to apparent satiation at 8:00 and 18:00 (Fu et al., 2017). Daily removal of residual feed and feces, together with water exchange with fresh aerated tap water, was performed to maintain optimal survival conditions (Cheng et al., 2008). Dead crabs were immediately removed for bacterial isolation and identification according to Liu et al. (2024) to confirm whether the mortality was caused by the isolate. Mortality of crabs in each group was recorded daily, and the LD_50_ value was estimated using the modified Kärber’s method (Zhang et al., 2022b).

#### 2.8.3 Protection against *Pseudomonas aeruginosa* infection assay

Prior to this assay, suspensions of the isolate and *P. aeruginosa* strain HX-1 were freshly prepared as described above (see sections 2.2 and 2.6). The two suspensions were, respectively, diluted to a final volume of 200 mL with sterile distilled water to obtain 3.0 × 10^4^, 3.0 × 10^5^ and 3.0 × 10^6^ CFU/mL of the isolate and 2.5 × 10^7^ CFU/mL of *P. aeruginosa*. These dilutions were added separately to 1.0 kg of the sterile-processed basal diet (Hongxiang Feed Biotech. Co., Ltd., Jiangsu, China), and each mixture was thoroughly blended in a drum mixer for 15 min and subsequently air-dried under sterile conditions. Following CFU enumeration on NA plates using serial 10-fold dilutions with sterile normal saline, the isolate-supplemented diets contained 6.0 × 10^3^, 6.0 × 10^4^, and 6.0 × 10^5^ CFU/g diet of the isolate, while the *P. aeruginosa*-supplemented diet contained 5.0 × 10^6^ CFU/g diet of *P. aeruginosa*. In total, 240 crabs were randomly assigned to 24 glass aquaria (60 cm × 40 cm × 40 cm), with 10 crabs per aquarium, as recommended by Cao et al. (2022), and no blinding was performed. Each aquarium served as an experimental unit and was supplied with three tiles at the bottom and 10 L of aerated tap water at 28 °C. Water quality parameters were maintained at pH 7–8, dissolved oxygen ≥ 6.0 mg/L, and total ammonia ≤ 0.2 mg/L, and monitored daily during the experiment. The aquaria were randomly divided into one negative control group, three safety control groups, one challenge control group, and three treatment groups, with three replicate aquaria per group. Crabs in the safety control and treatment groups were, respectively, fed with 6.0 × 10^3^, 6.0 × 10^4^, and 6.0 × 10^5^ CFU/g diet of the isolate-supplemented diets. In contrast, crabs in the negative control and challenge control groups were fed the sterile-processed basal diet under identical experimental conditions. Following 20 days of feeding, crabs in challenge control and treatment groups were orally challenged with the *P. aeruginosa*-supplemented diet for 7 days, whereas crabs in the negative control and safety control groups received no pathogen challenge. During the challenge test, crabs were fed at 8:00 and 18:00 up to apparent satiation with 3% of the total body weight (Fu et al., 2017), and kept at 28 °C under a 12 h light:12 h dark cycle (Speights and McCoy, 2017). Daily removal of residual feed and feces, together with water exchange with fresh aerated tap water, was carried out to assure optimal survivals (Cheng et al., 2008). Mortality in each group was recorded daily throughout the challenge period. The relative percent survival (RPS) was calculated using the following formula (Souza et al., 2017).

### 2.9 Statistical analysis

All data are presented as mean ± standard deviation (SD), and analyzed using one-way analysis of variance (ANOVA) with Dunnett’s *post hoc* test in SPSS 19.0 software (SPSS, Inc., Chicago, IL, United States). Mortality data were analyzed using Kaplan–Meier method and log rank test in the SPSS 18.0 software (SPSS, Inc.). Statistical significance is defined as *p* < 0.05.

## 3 Results

### 3.1 Bacterial isolation and identification

**Figure 1:**
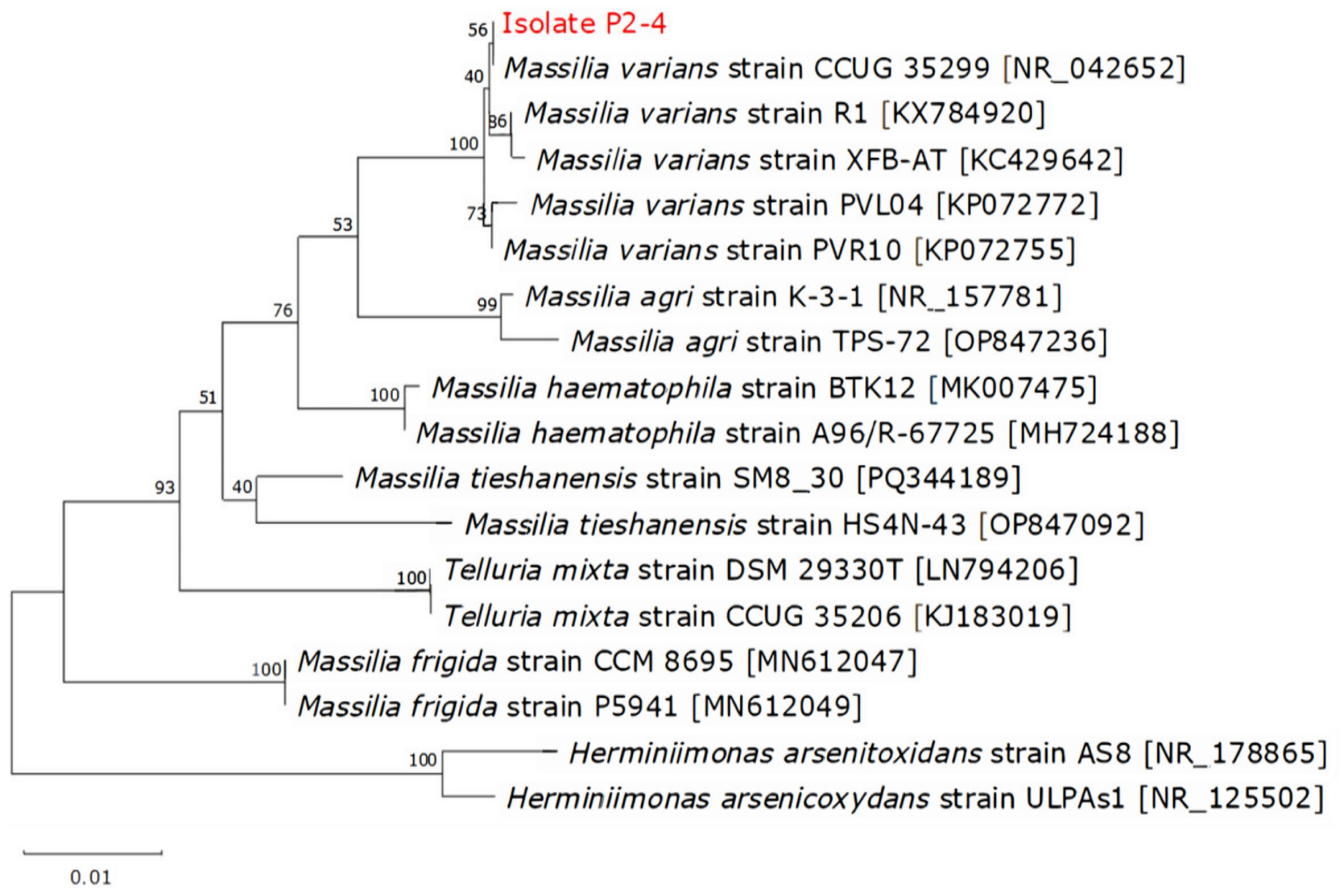
The 16S rRNA phylogenetic tree of isolate P2-4 and 17 known bacteria constructed using the neighbor-joining method. The bootstrap values (%) are shown beside the clades, accession numbers are indicated beside the names of strains, and scale bars represent distance values.

**Figure 2:**
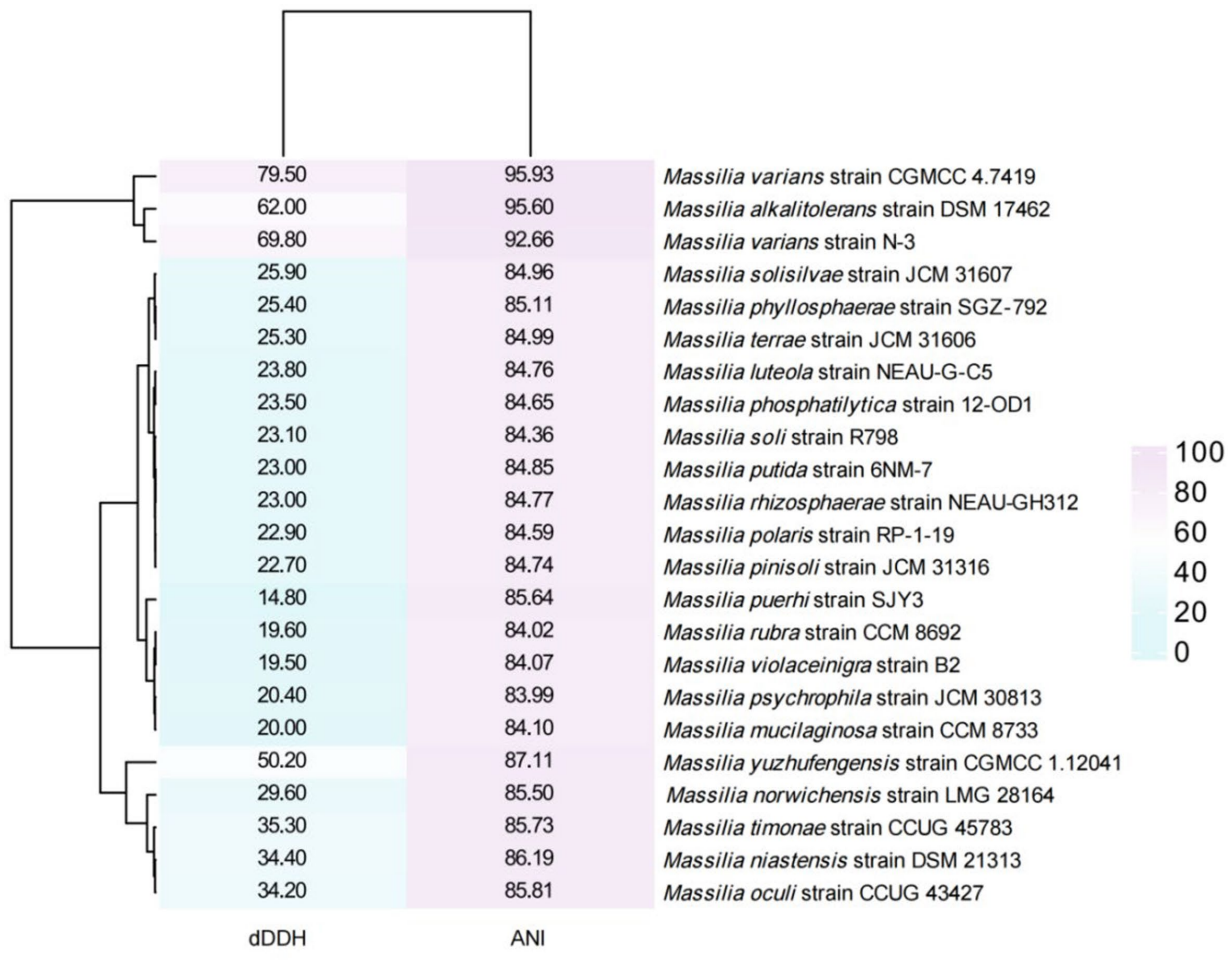
Heat map analysis of the genetic similarity between isolate P2-4 and other Massilia species based on the whole genome sequence. ANI, average nucleotide identity; dDDH, digital DNA– DNA hybridization.

A potential predatory isolate, designated P2-4, was isolated from aquaculture sediment, and it exhibited clear and visible plaques on the double-layer agar plate (Supplementary Figure S1), similar to those shown by *bdellovibrios* (Cao et al., 2007). It grew well on nutrient agar, but was unable to grow on TCBS agar. Its partial 16S rRNA gene and whole-genome sequences were, respectively, deposited in the NCBI BioSample and GenBank databases under accession nos. PV612026, SAMN53514402 and CM132631. Sequence analysis showed that it shared a similarity of 97 to 99% with *M. varians* strains available in the GenBank database, and was further confirmed as a *M. varians* strain based on the phylogenetic analysis (Figure 1). In addition, isolate P2-4 harbored one chromosome and one plasmid (Figure S2). Its whole genome size was 5,971,490 bp, with a completeness of 99.95% and an average GC content of 65.24%. Totally, 75 tRNAs, 21 rRNAs, 34 sRNAs, 5,238 CDS, 102 repeats, 1 prophage, 20 GI, and 23 IS were predicted in its genome (Table 1). Furthermore, the ANI and dDDH values between isolate P2-4 and *M. varians* type strain CGMCC 4.7419% were 95.93% and 79.50%, respectively. These values surpassed the species delineation thresholds of ANI (95%) and dDDH (70%) (Figure 2), further supporting isolate P2-4 as a *M. varians* strain.

**Table 1:** Genomic features of isolate P2-4.

Feature	Genome
Whole genome size (bp)	5,971,490
Chromosome size (bp)	5,657,168
Plasmid size (bp)	314,322
Plasmid	1
Chromosome	1
GC average content (%)	65.24
Number of CDSs	5,238
Gene average length (bp)	1017.87
Number of tRNAs	75
Number of 5S rRNAs	7
Number of 16S rRNAs	7
Number of 23S rRNAs	7
sRNAs	34
Repeated regions (%)	0.23
Number of repeats	102
Number of GI	20
Number of prophages	1
Number of IS	23

GC, guanine–cytosine; CDS, coding sequences; GIs, genomic islands; IS, insertion sequence.

Phenotypically, isolate P2-4 was Gram-negative rod-shaped and formed round, slightly convex, smooth, moist, regular-edged, and pale-yellow colonies on the NA plate (Supplementary Figure S3). It was positive for assimilation of glucose, maltose, and malate, and negative for nitrate reduction, indole production, glucose fermentation, arginine hydrolase, urease, *β*-glucosidase, and assimilation of arabinose, mannose, mannitol, N-acetyl-glucosamine, gluconate, capric acid, adipic acid, citrate, and phenylacetic acid (Table 2). These findings indicated that isolate P2-4 showed only 76.47% similarity to the reference strains of *M. varians* (Table 2) and differed from the reference strains in gelatinase production as well as glucose and arabinose fermentation, which underscored the inherent phenotypic diversity among *M. varians* strains.

**Table 2:** Phenotypic traits of isolate P2-4.

Test	Reaction	
	Isolate P2-4	*M.varians* ^a^
Nitrate reduction	−	−
Indole production	−	−
Glucose fermentation	−	−
Arginine hydrolase	−	ND
Urease	−	−
β-Glucosidase	−	−
Gelatinase	−	+
β-Galactosidase	−	−
Assimilation of glucose	+	−
Arabinose	−	+
Mannose	−	−
Mannitol	−	−
N-acetyl-D- (+)-glucosamine	−	ND
Maltose	+	+
Gluconate	−	−
Capric acid	−	ND
Adipic acid	−	−
Malate	+	±
Citrate	−	−
Phenylacetic acid	−	+

+, positive reaction; −, negative reaction; ±, positive or negative reactions; ND, not done. a, data previously reported (Cho et al., 2017; Chaudhary and Kim, 2017; Weon et al., 2010).

### 3.2 Bacterial virulence and antibacterial-related genes

Isolate P2-4 possessed no virulence genes based on VFDB annotation. Genomic analysis against the COG and KEGG databases identified 19 antibacterial-related genes, such as those encoding pilus fiber forming protein PilA (*pilA*), pilus assembly ATPase PilB (*pilB*), pilin assembly protein PilC (*pilC*), prepilin peptidase PilD (*pilD*), prepilin leader peptidase PilF (*pilF*), signal transduction response regulator pilG (*pilG*), signal transduction response regulator PilH (*pilH*), pilus assembly protein PilM (*pilM*), pilus biogenesis protein PilO (*pilO*), pilus assembly protein PilP (*pilP*), pilus secretin PilQ (*pilQ*), pilus biogenesis protein PilS (*pilS*), pilus transcriptional regulator (*pilR*), pilus twitching motility protein PilT (*pilT*), lytic transglycosylase MltA (*mltA*), lytic transglycosylase MltB (*mltB*), lytic transglycosylase MltC (*mltC*), lytic transglycosylase MltD (*mltD*), and endopeptidase DacB (*dacB*) (Table 3). The presence of these functional genes may contribute to the antibacterial capacity of isolate P2-4.

**Table 3:** Potential bacteriolysis-related genes in isolate P2-4.

Gene	Product	Identity (%)	Coverage (%)	E-value
*pilA*	Pilus fiber-forming protein PilA	100	95.31	1.3e^−31^
*pilB*	Pilus assembly ATPase PilB	99.7	99.83	0
*pilC*	Pilin assembly protein PilC	98.3	99.7	7.21e^−276^
*pilD*	Prepilin peptidase PilD	99.7	98.97	7.02e^−201^
*pilF*	Prepilin leader peptidase PilF	92.9	98.51	3.46e^−165^
*pilG*	Signal transduction response regulator PilG	100	99.16	6.95e^−77^
*pilH*	Signal transduction response regulator PilH	100	96.9	5.76e^−80^
*pilM*	Pilus assembly protein PilM	99.7	99.72	1.01e^−244^
*pilO*	Pilus biogenesis protein PilO	99.5	99.55	9.83e^−149^
*pilP*	Pilus assembly protein PilP	98.4	99.45	2.41e^−123^
*pilQ*	Pilus secretin PilQ	98.5	99.32	0
*pilS*	Pilus biogenesis protein PilS	98.2	99.82	0
*pilR*	Pilus transcriptional regulator PilR	98.6	99.79	6.43e^−312^
*pilT*	Pilus twitching motility protein PilT	100	99.71	2.38e^−244^
*mltA*	Lytic transglycosylase MltA	97.7	95.62	3.47e^−256^
*mltB*	Lytic transglycosylase MltB	97.5	99.75	1.42e^−265^
*mltC*	Lytic transglycosylase MltC	100	94.98	9.92e^−189^
*mltD*	Lytic transglycosylase MltD	99.3	99.78	7.18e^−308^
*dacB*	Endopeptidase DacB	96.7	99.19	0

### 3.3 Bacterial toxic metabolite production

Isolate P2-4 did not produce toxic metabolites, such as hemolysin, hydrogen sulfide, nitrite, and ammonia (Table 4). Meanwhile, the control strain *R. azotoformans* strain SY5 also failed to produce these toxic metabolites, consistent with the previous data from Cao et al. (2023). These findings indicated the reliability of the toxic metabolite testing result with isolate P2-4, and suggested the non-toxic property of isolate P2-4.

**Table 4:** The toxic metabolites production of isolate P2-4.

Toxic metabolite	Isolate P2-4	*R. azotoformans* strain SY5
Haemolysin	−	−
Hydrogen sulfide	−	−
Nitrite	−	−
Ammonia	−	−

−, negative reaction.

### 3.4 Bacterial antibacterial spectrum

**Figure 3:**
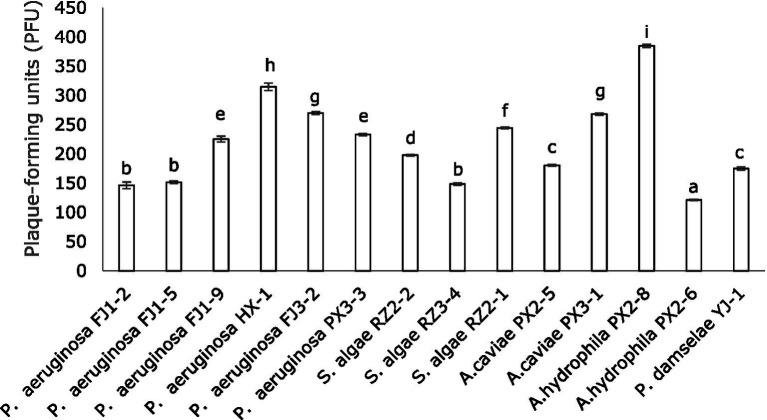
Antibacterial activity of isolate P2-4 against bacterial pathogens. Values are presented as mean ± SD (*n* = 3). Data represented by different letters are significantly different at *p* < 0.05.

Isolate P2-4 showed potential antibacterial activity against *P. aeruginosa*, *S. algae, A. caviae, A. hydrophila,* and *P. damselae*. The maximum plaque count (385 PFU) was recorded in *A. hydrophila* strain PX2-8, which was significantly higher (*p* < 0.05) than those for *P. aeruginosa* strain HX-1 (315 PFU)*, P. aeruginosa* strain FJ3-2 (270 PFU), *A. caviae* strain PX3-1 (268 PFU), *S. algae* strain RZ2-1 (244 PFU), *P. aeruginosa* strain FJ1-9 (225 PFU)*, P. aeruginosa* strain PX3-3 (233 PFU)*, S. algae* strain RZ2-2 (198 PFU), *A. caviae* strain PX2-5 (180 PFU), *P. damselae* strain YJ-1 (175 PFU), *P. aeruginosa* strain FJ1-5 (151 PFU), *S. algae* strain RZ3-4 (148 PFU), *P. aeruginosa* strain FJ1-2 (146 PFU), and *A. hydrophila* strain PX2-6 (121 PFU) (Figure 3).

### 3.5 *In vitro* bacterial antibacterial effect

**Figure 4:**
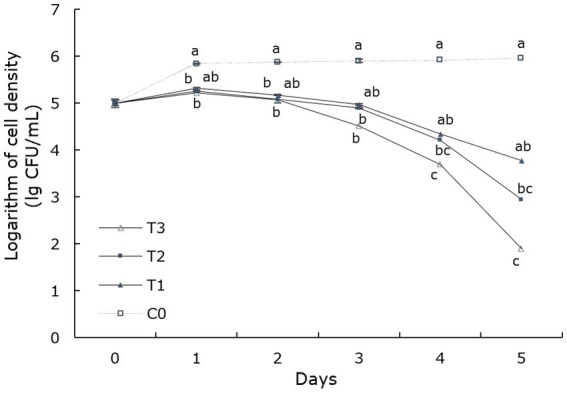
Effect of isolate P2-4 on the growth of *P. aeruginosa*. C0, *P. aeruginosa* only; T1, *P. aeruginosa* + 1.0 × 10^3^ CFU/mL of isolate P2-4; T2, *P. aeruginosa* + 1.0 × 10^4^ CFU/mL of isolate P2-4; T3, *P. aeruginosa* + 1.0 × 10^5^ CFU/mL of isolate P2-4. Values are presented as mean ± SD (*n* = 3). Data represented by different letters at the same time points are significantly different at *p* < 0.05.

Compared with the control, the growth of *P. aeruginosa* was significantly inhibited after treatment with isolate P2-4. The cell density logarithms of *P. aeruginosa* were significantly reduced by 36.85% (*p* < 0.05), 50.89% (*p* < 0.05), and 68.17% (*p* < 0.05) following 5 days of co-culture with isolate P2-4 at 1.0 × 10^3^, 1.0 × 10^4^, and 1.0 × 10^5^ CFU/mL (Figure 4). The corresponding bacteriostatic rates against *P. aeruginosa* reached 99.35%, 99.90%, and 99.99%, respectively. These findings suggested a significant antibacterial effect of isolate P2-4 against *P. aeruginosa*.

### 3.6 Bacterial pathogenicity and protection against *Pseudomonas aeruginosa* infection

**Figure 5:**
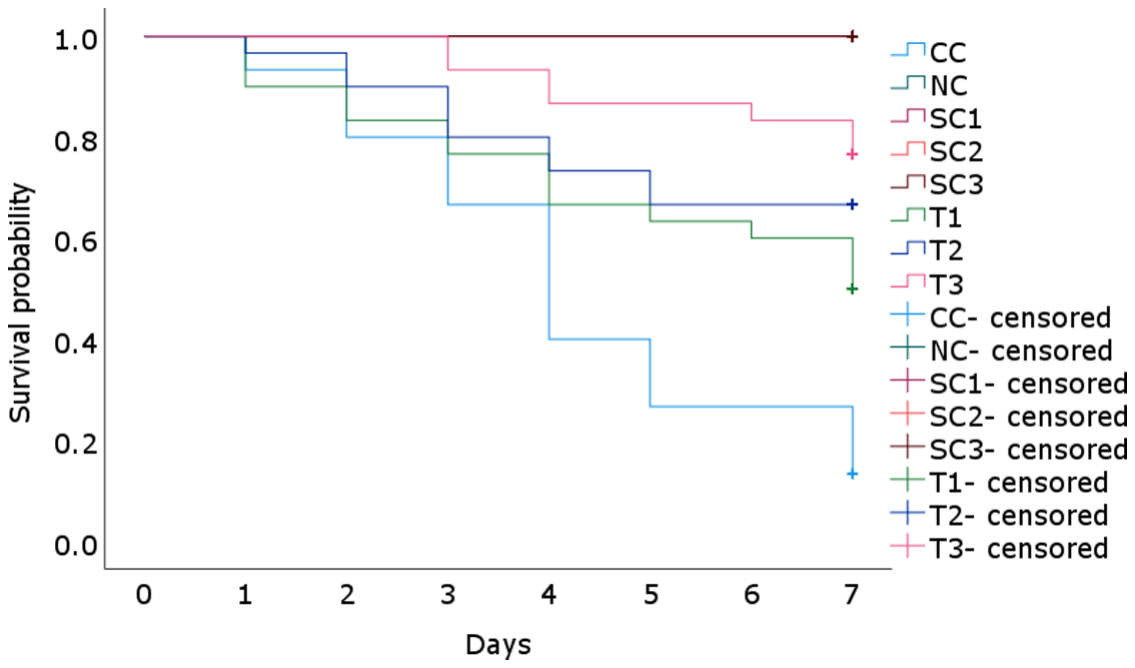
Protective effect of isolate P2-4 against *P. aeruginosa* infection in *E. sinensis*. NC, negative control crabs fed basal diets with no bacterial challenge; SC1, crabs fed diets supplemented with 6.0 × 10^3^ CFU/g diet of isolate P2-4 with no bacterial challenge; SC2, crabs fed diets supplemented with 6.0 × 10^4^ CFU/g diet of isolate P2-4 with no bacterial challenge; SC3, crabs fed diets supplemented with 6.0 × 10^5^ CFU/g diet of isolate P2-4 with no bacterial challenge; CC, challenge control crabs fed basal diets and challenged with *P. aeruginosa*; T1, crabs fed 6.0 × 10^3^ CFU/g diet of isolate P2-4-supplemented diets and challenged with *P. aeruginosa*; T2, crabs fed 6.0 × 10^4^ CFU/g diet of isolate P2-4-supplemented diets and challenged with *P. aeruginosa*; T3, crabs fed 6.0 × 10^5^ CFU/g diet of isolate P2-4-supplemented diets and challenged with *P. aeruginosa*.

Neither mortality nor pathological signs were observed in crabs from the control group and treatment groups treated with 5.0 × 10^4^ ~ 5.0 × 10^8^ CFU/mL of isolate P2-4 (data not shown), which indicated that the 7-day acute LD_50_ value of isolate P2-4 exceeded 5.0 × 10^8^ CFU/mL in *E. sinensis*. These findings indicated that isolate P2-4 was an avirulent strain toward *E. sinensis* based on the acute LD_50_ avirulent threshold (≥ 10^7^ CFU/mL). Besides, crabs fed diets supplemented with isolate P2-4 exhibited significant improvement in survival against *P. aeruginosa* challenge. Following 7 days of oral challenge with *P. aeruginosa*, mortality rates were significantly reduced by 36.67% (*p* < 0.05), 53.34% (*p* < 0.05), and 63.34% (*p* < 0.05) in crabs fed diets supplemented with 6.0 × 10^3^, 6.0 × 10^4^, and 6.0 × 10^5^ CFU/g diet of isolate P2-4 as compared to the challenge control (Figure 5). The corresponding RPS values for isolate P2-4 at 6.0 × 10^3^, 6.0 × 10^4^, and 6.0 × 10^5^ CFU/g diet were 42.31, 61.54 and 73.08%, respectively. Meanwhile, 100% survival was recorded for crabs from the negative control and non-challenged isolate P2-4-treated groups. The disease sign of hepatopancreatic decoloration was observed in challenge crabs (Supplementary Figure S4), consistent with that in naturally infected crabs as described by He et al. (2026). The pathogenic *P. aeruginosa* was successfully re-isolated from all dead crabs and was confirmed via phenotypic and molecular identification (data not shown). These findings indicated that isolate P2-4 conferred significant protection to crabs against *P. aeruginosa* infection.

## 4 Discussion

Recently, environmentally friendly control agents have been proposed to control pathogenic *P. aeruginosa* in aquaculture, such as the use of *Melaleuca alternifolia* essential oil, Yucca extract, *Chaetomorpha linum* extract, grape pomace flour, bacteriophages, *Bacillus coagulans*, *Padina boergesenii*, and levamisole (Koudou et al., 2020; Baldissera et al., 2019; El-Gammal et al., 2025; Ji et al., 2022; Naena, 2020; Ragunath and Ramasubramanian, 2022; Souza et al., 2017; Thanigaivel et al., 2023). Yet no reports are available on *M. varians* strains as biocontrol agents against pathogenic *P. aeruginosa* in aquaculture. In this study, we identified a *M. varians* isolate P2-4 as a potential candidate for a probiotic against crab-pathogenic *P. aeruginosa* through comprehensive analyses of genome-based antibacterial-related genes, virulence genes, toxic metabolites, pathogenicity, and *in vitro* and *in vivo* antibacterial effects. To our knowledge, this is the first report describing a *M. varians* strain as a potential biocontrol agent against pathogenic *P. aeruginosa* in aquaculture.

In contrast to obligate predators such as *Bdellovibrio* and like organisms (BALOs), non-obligate predators such as members of *Myxococcales*, *Streptomycetaceae*, and *Cytophagia* have a broader distribution in water ecosystems (Hungate et al., 2021) and can grow on nutrient agar (Seccareccia et al., 2015). Accordingly, we isolated potential non-obligate predatory bacteria from aquaculture sediment and quantified isolate P2-4 using nutrient agar. However, isolate P2-4 showed no growth on TCBS agar. Thus, TCBS agar was used to quantify the cell density of *P. aeruginosa* co-cultured with isolate P2-4.

Given that 16S rRNA gene sequencing and phenotypic characterization analyses are insufficient for accurate differentiation of *Massilia* species, whole genome sequencing is recommended for precise species identification (Baek et al., 2022). In this study, whole genome-based analysis yielded 95.93% ANI identity and 79.50% dDDH similarity to *M. varians* type strain CGMCC4.7419, taxonomically suggesting isolate P2-4 as a *M. varians* strain based on the species delineation thresholds of 95% ANI and 70% dDDH (Candeliere et al., 2021). However, isolate P2-4 exhibited only 76.47% phenotypic identity to *M. varians* reference strains and differed from clinical isolates of *M. varians* in arabinose and malate assimilation (Cho et al., 2017). The differences in genomic and biochemical traits between potential probiotic and clinical *M. varians* strains suggest intraspecific heterogeneity in this species.

Antibacterial-related genes are of great importance to the antibacterial capability of predatory bacteria (Oyedara et al., 2018). For example, type IV pili-encoding genes, such as *pilA*, *pilT, pilQ*, *pilM*, *pilS*, and *pilR*, are associated with adherence to and invasion of prey cells, and thus play a crucial role in antibacterial activity of predatory bacteria (Evans et al., 2007; Mahmoud and Koval, 2010). Genes encoding lytic transglycosylases and endopeptidases, such as *mltA*, *mltB*, *mltC*, *mltD*, and *dacB*, are involved in hydrolysing polysaccharide linkages in murein and attacking the peptide cross-links (Bernhardt and De Boer, 2004), which result in relaxation of the peptidoglycan layer and elimination of the prey cell septum obstruction (Dörr, 2023; Lerner et al., 2012). In this study, isolate P2-4 harbored not only the above antibacterial genes (*pilA*, *pilT, pilQ*, *pilM*, *pilS*, *pilR, mltA*, *mltB*, *mltC*, *mltD*, and *dacB*) responsible for antibacterial capability and prey cell elimination but also other type IV pili-encoding genes, such as *pilB*, *pilC*, *pilD*, *pilF*, *pilG*, *pilH*, *pilO*, and *pilP*, which could potentially contribute to its antibacterial activity (Mahmoud and Koval, 2010; Manetti and Spadafina, 2014). The presence of these antibacterial-related genes provides a genetic explanation for the antibacterial capacity of isolate P2-4.

Pathogenicity analysis of potential probiotic strains in aquaculture should be conducted prior to practical application (Verschuere et al., 2000). Whole-genome-based gene mining is strongly recommended for assessing the pathogenicity of probiotics (Wang et al., 2021). Acute LD_50_ also serve as a reliable indicator to assess the pathogenicity of probiotics in aquaculture (Turnip et al., 2018; Zhou et al., 2000). Strains with an acute LD₅₀ above 10^7.0^ CFU/mL are classified as avirulent. In our study, isolate P2-4 carried no virulence genes through genome blast against the VFDB database and showed an acute LD_50_ value of above 5.0 × 10^8^ CFU/mL in *E. sinensis*. These findings suggested that isolate P2-4 possessed no pathogenic potential. Furthermore, isolate P2-4 failed to produce toxic hemolysin, hydrogen sulfide, nitrite, and ammonia that could cause systemic damage and toxic responses in crabs (Abd-El-Malek, 2017; Koo et al., 2005; Xu et al., 2014), further supporting it as a potential non-toxic strain. However, it should be noted that *M. varians* strains have occasionally been recovered from human clinical specimens (Cho et al., 2017; Kampfer et al., 2008). A similar situation is observed for *Enterococcus faecalis* and *Weissella confuse*, which are regarded as potential probiotics yet may opportunistically cause infections in humans and animals (De Almeida et al., 2018; Fairfax et al., 2014). Thus, conspecific strains with probiotic traits still represent promising candidates for the pathogen control and disease treatment (Chauhan and Singh, 2019). Before large-scale application of isolate P2-4, further comprehensive safety assessment must be performed, such as antimicrobial resistance gene and plasmid-associated resistance detection, mobile genetic element screening, antibiotic susceptibility testing, longer-term oral safety evaluation, histopathological analysis, and potential impact assessment on non-target microbiota.

Obligate predatory bacteria are considered promising biocontrol agents since they can prey upon and kill a broad spectrum of multiple bacterial pathogens (Damron and Barbier, 2013; Melander et al., 2018). For instance, *Bdellovibrio* sp. isolate T1 displays antibacterial activity against 45.45% of the 11 test prey strains (Ezzedine et al., 2020). *Bdellovibrio* sp. isolate YBD-1 exhibits antibacterial activity against 92.31% of the 13 test prey strains (Xi et al., 2024). In this study, isolate P2-4 exhibited potential antibacterial activity against all test aquatic pathogens, such as *P. aeruginosa*, *A. caviae*, *A. hydrophila, P. damselae*, and *S. algae*, which pose a significant threat to aquaculture (Dehkordi et al., 2022; Lattos et al., 2022; Tao et al., 2026; Zago, 2020). This suggests higher antibacterial activity of isolate P2-4 than the obligate *Bdellovibrio* sp. isolates T1 and YBD-1 (Ezzedine et al., 2020; Xi et al., 2024). The broad-spectrum inhibitory capacity of isolate P2-4 suggests its potential as a probiotic candidate to control pathogenic *P. aeruginosa*, *A. caviae, A. hydrophila, P. damselae*, and *S. algae* in aquaculture.

*Pseudomonas aeruginosa* is an emerging but underestimated bacterial pathogen in *E. sinensis* aquaculture (He et al., 2026). To assess the application potential of isolate P2-4, this study examined its *in vitro* and *in vivo* antibacterial effects against the *E. sinensis*-pathogenic *P. aeruginosa.* The findings indicated that isolate P2-4 at 1.0 × 10^3^ to 1.0 × 10^5^ CFU/mL displayed bacteriostatic rates of 99.35% to 99.99% against the crab-pathogenic *P. aeruginosa.* This potent inhibitory effect may stem from multiple type IV pili-related *pil* genes, which may facilitate its antibacterial performance in liquid co-culture systems (Evans et al., 2007). Furthermore, isolating P2-4 at 6.0 × 10^3^ to 6.0 × 10^5^ CFU/g diet also led to RPS of 42.31 to 73.08% against *P. aeruginosa* infection in *E. sinensis.* This protective effect might be attributed to the reduction of the pathogen loads due to the antibacterial action of isolate P2-4 (Cao et al., 2014a).

## 5 Conclusion

This study, for the first time, revealed the *M. varians* isolate (P2-4) as a potential biocontrol agent against *P. aeruginosa* in aquaculture. The absence of virulence genes, abundant antibacterial-related genes, non-pathogenic and non-toxic properties, and prominent *in vitro* and *in vivo* antibacterial efficacy of isolate P2-4 collectively indicate that this strain is a promising probiotic candidate against pathogenic *P. aeruginosa* in aquaculture.

## Data Availability

The datasets presented in this study can be found in online repositories. The names of the repository/repositories and accession number(s) can be found in the article/Supplementary material.

## Footnotes

1
https://blast.ncbi.nlm.nih.gov/Blast.cgi

2 VFDB, v. 20,230,407, http://www.mgc.ac.cn/VFs/
