# Supplementary material for: Massilia varians P2-4: a potential biocontrol agent against pathogenic Pseudomonas aeruginosa in Eriocheir sinensis
Source: Front Microbiol. 2026 Jun 30;17:1863139. doi: 10.3389/fmicb.2026.1863139 (PMC13364903; doi:10.3389/fmicb.2026.1863139)
Supplement: Supplementary file 1 [file Table_1.DOC]

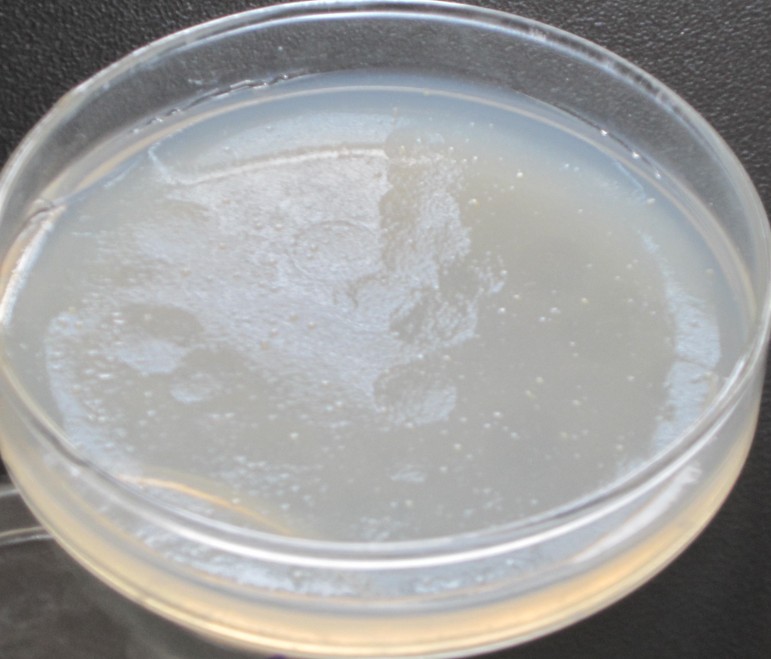


**Figure S1.** The plaques of isolate P2-4 formed on the double-layer agar plate after 5 days of incubation at 30°C, using *P. aeruginosa* as the prey bacterium. The arrow shows a clear and visible plaque.


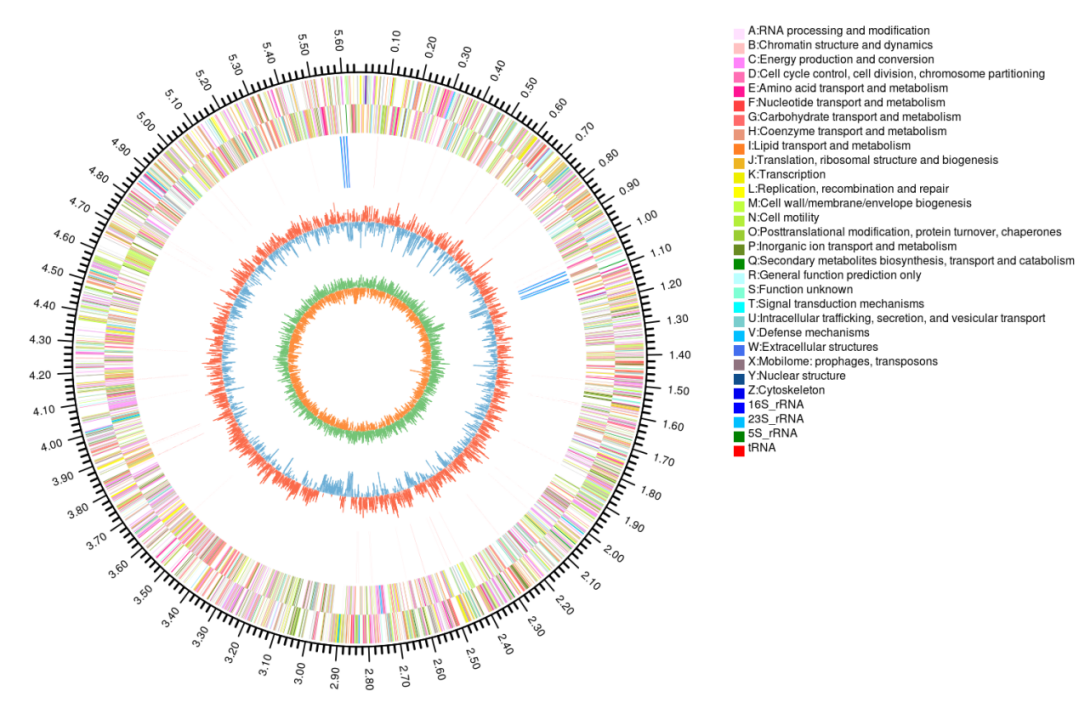


A


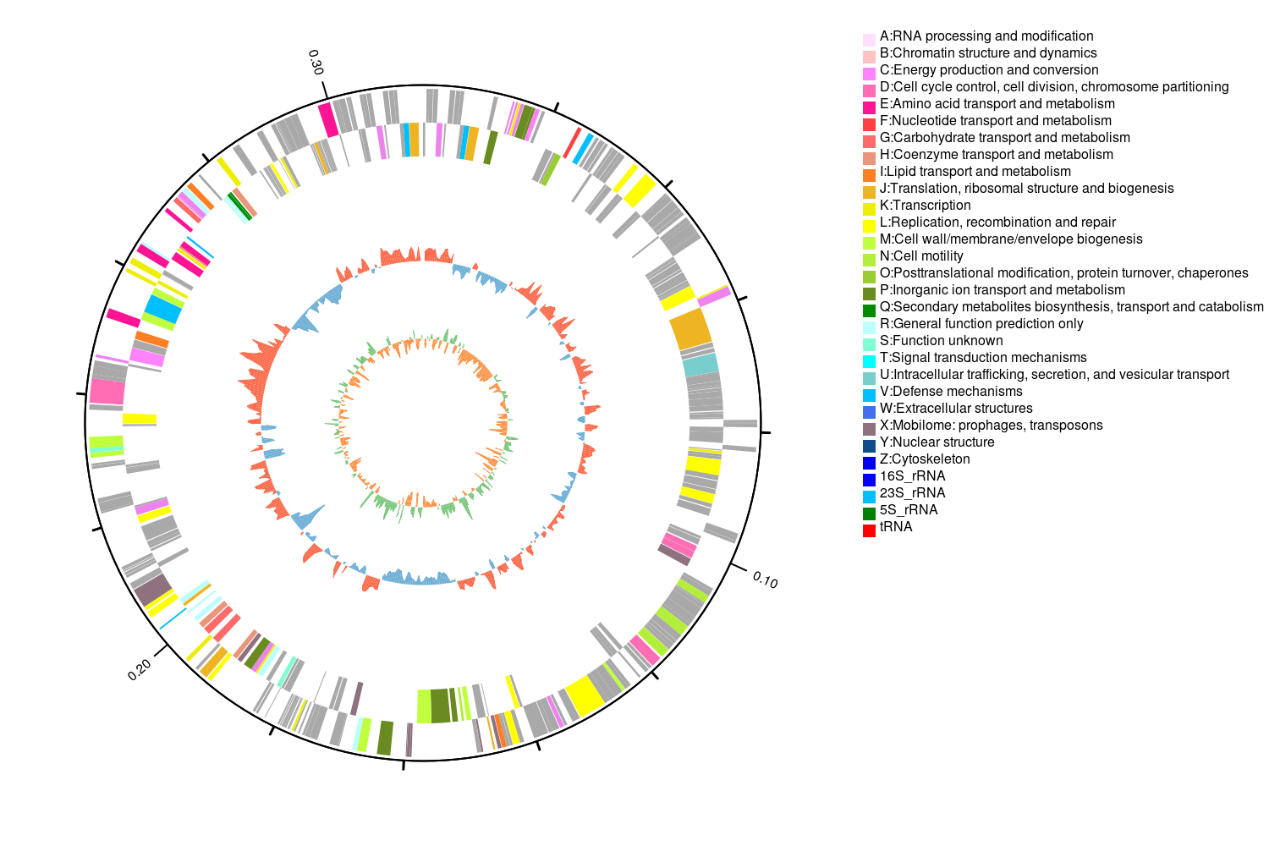


B

**Figure S2.** Circular complete genome map of isolate P2-4. (A) Circular compete genome map of chromosome. (B) Circular compete genome map of plasmid. From outside to inside, the maps show (1) the size of the complete genome, (2-3) forward and reverse CDSs with different colours representing different COG functional classifications (right-top of map), (4) rRNA and tRNA, (5) GC content and (6) GC skew value. Abbreviations: CDSs, coding sequences; GC, guanine-cytosine; rRNA, ribosomal RNA; tRNA, transfer RNA; COG, Clusters of Orthologous Groups.


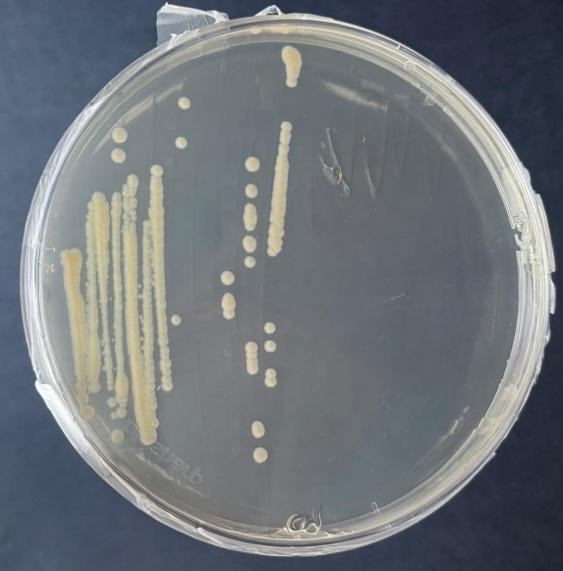

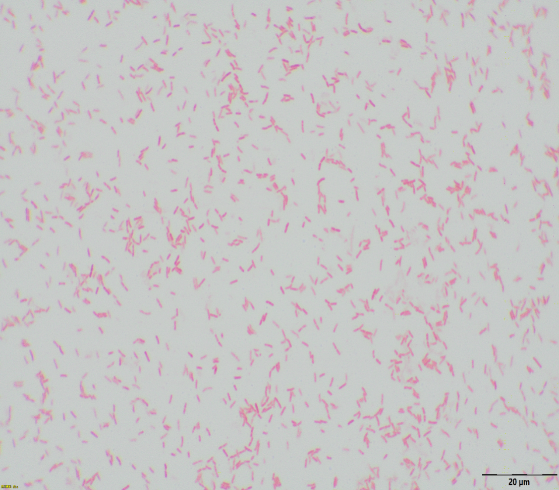


B

A

**Figure S3.** Morphological feature of isolate P2-4. (A) Colony morphology of isolate P2-4 cultured on a nutrient agar plate at 30 °C for 24 h. The arrow shows a round, slightly convex, smooth, moist, regular-edged, and pale yellow colony. (B) Microscopic morphology of isolate P2-4 (100×). The arrow shows a rod-shaped cell.


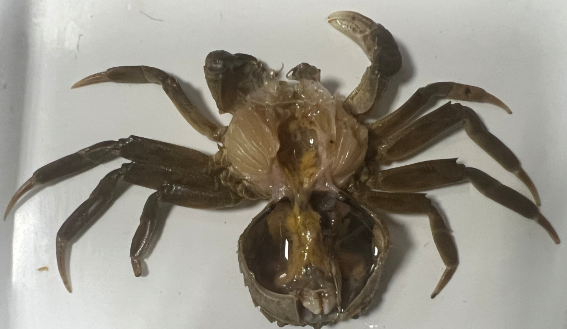


**Figure S4.** The gross sign of challenged crabs. Arrow shows the hepatopancreatic decoloration.
